# DPPZ–Naphthalimide Conjugates as G-Quadruplex DNA Targeting Scaffolds: Design, Synthesis and Biomolecular Interaction Studies

**DOI:** 10.3390/ph19040575

**Published:** 2026-04-02

**Authors:** Ufuk Yildiz, Özge Gökçek

**Affiliations:** Chemistry Department, Faculty of Science, Zonguldak Bülent Ecevit University, 67100 Zonguldak, Türkiye; ozgegokcek@gmail.com

**Keywords:** G-quadruplex targeting, DPPZ, naphthalimide, DNA binding ligands, anticancer agents

## Abstract

**Background:** Guanine-rich DNA regions can fold into G-quadruplex (G4) structures, which are prevalent in telomeres and oncogene promoters, making them attractive targets for anticancer therapeutics. Small molecules capable of selectively stabilizing G4 DNA can disrupt telomerase activity and oncogene expression, offering a promising strategy for cancer intervention. **Methods:** A rationally designed series of DPPZ–anhydride-conjugated ligands (**1** and **2**) and their corresponding quaternized derivatives (**1-q** and **2-q**) were synthesized to investigate the combined effects of π-extension, bromine substitution, and cationic modification on DNA recognition. The synthetic strategy relied on the incorporation of a highly planar DPPZ–anhydride scaffold to enhance π-surface area, followed by selective quaternization to introduce permanent positive charge and reinforce electrostatic interactions with the DNA backbone. All compounds were fully characterized by NMR and spectroscopic methods. The DNA-binding properties of the ligands were systematically evaluated toward duplex (ds-DNA) and G-quadruplex (G4-DNA) structures using UV–Vis absorption titration, fluorescence intercalator displacement (FID) assays, and competitive dialysis experiments. Quaternization markedly enhanced intrinsic binding constants and significantly reduced DC_50_ values, particularly for G4-DNA. While bromine substitution increased overall binding affinity, it did not substantially improve topology selectivity. Among the series, compound **1-q** exhibited the most favorable balance between affinity and G4 selectivity. **Results:** The interaction of the compounds with BSA was quantified using Stern–Volmer quenching constants, which demonstrated a clear trend of enhanced quenching efficiency upon modification. The binding strength followed a descending order of **1-q** > **2-q** > **1** > **2**, highlighting the superior performance of the first series over the second. These findings indicate that the structural features of **1-q** facilitate a more robust interaction within the hydrophobic pockets of the protein. **Conclusions:** Overall, the results demonstrate that strategic π-conjugation combined with electrostatic reinforcement provides an effective approach for the development of topology-selective DNA-binding ligands.

## 1. Introduction

Cancer development is closely associated with genomic instability and abnormal DNA structures, particularly G-quadruplex (G4) formations, which play key roles in telomere maintenance and oncogene regulation. Guanine-rich DNA regions can adopt G4 structures through Hoogsteen hydrogen bonding between guanine bases. These structures are commonly found in telomeres and oncogene promoter regions and play key roles in genome stability, replication, and transcription [1,2]. Stabilization of G4 DNA by small molecules has emerged as a promising anticancer strategy via telomerase inhibition and regulation of oncogene expression [3,4].

**Figure 1:**
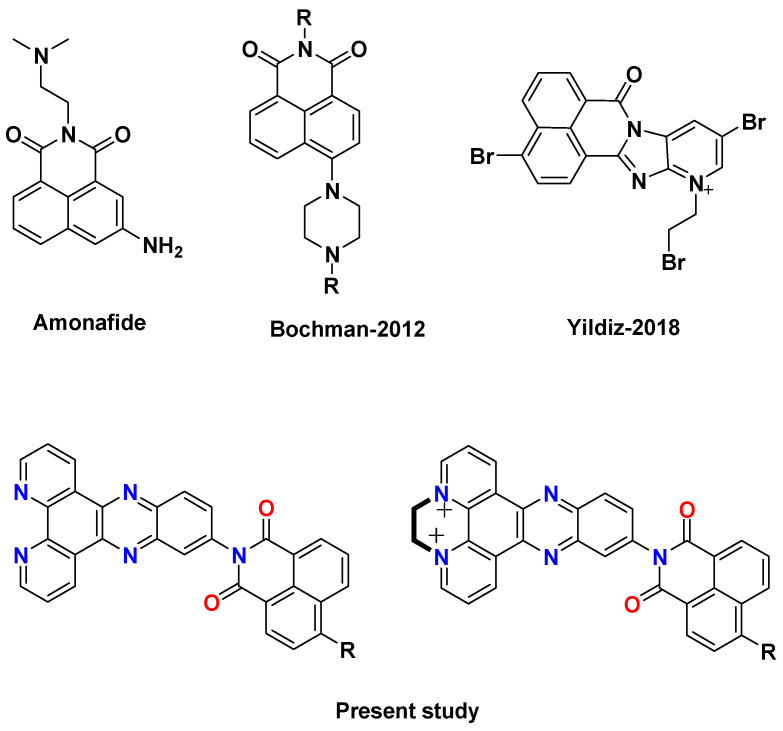
Structures of some naphthalimide derivatives in the literature [5,10] and the present work.

Naphthalimide and naphthalene diimide (NDI) derivatives have been widely studied as DNA-binding agents due to their planar aromatic structures, which enable π–π stacking with DNA base pairs or terminal G-quartets [5,6]. The presence of protonatable side chains enhances binding affinity and selectivity through electrostatic and hydrogen-bonding interactions [5,6]. In particular, 4-bromo-1,8-naphthalimide derivatives and tetrasubstituted NDIs have demonstrated strong G4 stabilization and binding affinity, as confirmed by spectroscopic and calorimetric studies [7,8,9,10,11] (Figure 1). Hybrid systems and metal complexes of these scaffolds have further improved selectivity and biological activity, including telomerase inhibition [12,13,14,15,16,17].

Dipyrido[3,2-a:2′,3′-c]phenazine (DPPZ) is another well-known DNA-binding ligand with a large aromatic surface that facilitates strong π–π interactions with G4 DNA, although its affinity for double-stranded DNA limits selectivity [18,19,20,21]. Conjugation of DPPZ with naphthalimide offers a promising strategy to combine multiple binding modes within a single molecule, potentially enhancing G4 selectivity and thermal stabilization [22,23,24].

In addition, DPPZ-based systems are capable of strong interactions with biomacromolecules such as Bovine Serum Albumin (BSA), owing to their extended aromatic surfaces and compatibility with hydrophobic binding pockets [25,26]. Understanding these interactions is important for predicting pharmacokinetic behavior and bioavailability.

In conclusion, DPPZ–naphthalimide conjugates are next-generation ligand candidates capable of stronger and more selective binding to G4-DNA using both π–π stacking and side chain interactions. These properties make them a valuable platform for anticancer research on telomerase inhibition and oncogene regulation.

**Scheme 1:**
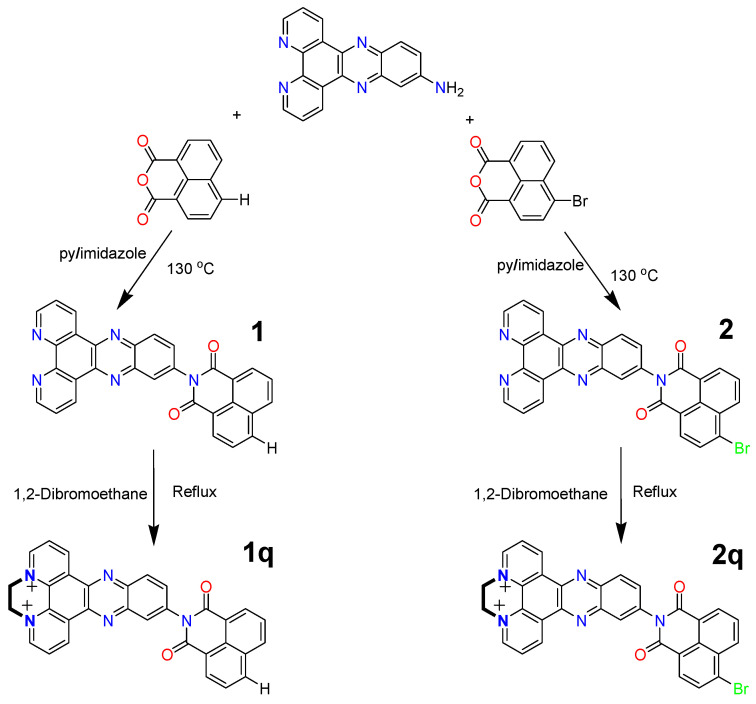
Synthetic route for compounds **1**, **1q**, **2** and **2q**.

This study was designed with the aim of synthesizing a DPPZ–naphthalimide-conjugated system with an extended aromatic surface and to investigate how functionalization at the 4-position, followed by quaternization, influences its selectivity toward G4 DNA (Scheme 1). For this purpose, two novel naphthalimide–DPPZ-conjugated ligands bearing bromine (-Br) and hydrogen (-H) substituents at the 4-position were synthesized (Figure 1). Subsequently, the ligands were quaternized through the DPPZ unit in order to enhance their water solubility and biological activity. The synthesized compounds were comprehensively characterized by FT-IR, ^1^H-NMR, ^13^C-NMR, and ESI-MS analyses, and their interactions with double-stranded DNA, G4-DNA and BSA were systematically investigated employing a range of biophysical methods.

## 2. Results

### 2.1. Synthesis of Compounds

The synthetic strategy used in the preparation of the DPPZ–naphthalimide conjugates aimed to create an extended π-conjugated framework to enhance potential biological activity and then target structural cationization.

The condensation of amino-DPPZ with 1,8-naphthalic anhydride and its bromo analog yielded compounds **1** and **2** via classical imidization under pyridine/imidazole conditions at elevated temperatures. FT-IR spectra clearly confirmed imide formation by the presence of strong carbonyl stretching bands around ~1705 cm^−1^ and the absence of anhydride carbonyl absorptions. Persistent C=N stretching bands (~1668 cm^−1^) indicate that the DPPZ core is preserved during imidization. ESI-MS data further supported the proposed molecular compositions, while ^1^H NMR spectra showed the expected aromatic proton distributions corresponding to the fused polyaromatic system. In the case of the brominated derivative (**2**), the increased molecular mass and characteristic isotopic contribution indicated bromination. In compound **1**, the ^1^H NMR spectrum (DMSO-*d*_6_ + TFA) exhibits characteristic downfield signals at δ 10.08 and 10.01 ppm (each d, 1H), which can be assigned to peri-position aromatic protons located adjacent to the imide carbonyl groups and electron-deficient phenazine nitrogens. The multiplet at δ 9.36 ppm (2H) corresponds to protons positioned ortho to the phenazine nitrogens within the DPPZ core, where electron withdrawal induces significant downfield shifts. Doublets observed at δ 8.79 (2H) and 8.63 ppm (1H) are consistent with aromatic protons on the extended phenanthroline-like framework of DPPZ. The singlet at δ 8.52 ppm (1H) can be attributed to a proton located on a relatively isolated aromatic carbon within the fused system, likely at the central phenazine ring. Additional doublets and multiplets between δ 8.45–7.93 ppm correspond to the remaining aromatic protons of the DPPZ and naphthalimide moieties. The overall aromatic pattern, consisting exclusively of signals in the 7.9–10.1 ppm range, is consistent with a highly conjugated and electron-deficient polyaromatic scaffold. For compound **2**, a similar aromatic distribution is observed. However, slight downfield shifts in selected signals (δ 9.61 and 9.56 ppm) reflect the additional electron-withdrawing effect of the bromine substituent on the naphthalimide ring. The structural integrity of the synthesized conjugate was further confirmed by ^13^C NMR spectroscopy (DMSO-*d*_6_). The spectrum displays resonances fully consistent with the proposed DPPZ–naphthalimide framework.

The structures of the DPPZ–naphthalimide conjugates were further confirmed by ^13^C NMR spectroscopy (DMSO-*d*_6_). Both compounds exhibit characteristic resonances consistent with an extended π-conjugated aromatic framework incorporating a naphthalimide unit.

For compound **1**, the ^13^C NMR spectrum shows aromatic carbon signals distributed between δ 121–149 ppm. Signals in the δ 123–132 ppm region are assigned primarily to protonated aromatic carbons (Ar–CH) within the DPPZ and naphthalimide rings. Resonances between δ 132–142 ppm correspond mainly to quaternary aromatic carbons located at ring fusion positions. More deshielded signals above δ 140 ppm are attributed to carbons adjacent to nitrogen atoms in the phenazine core, reflecting the electron-withdrawing nature of the heteroaromatic system. The imide carbonyl carbon appears at δ 165.15 ppm, which falls within the typical range for naphthalimide C=O carbons (162–166 ppm), confirming successful imide formation. The absence of additional carbonyl resonances in the 170–175 ppm region further supports complete conversion of the starting anhydride.

Compound **2** displays a very similar aromatic carbon distribution (δ 123.08–150.18 ppm), confirming the preservation of the conjugated backbone upon bromine substitution. However, subtle downfield shifts are observed for several aromatic carbons, particularly in the δ 140–150 ppm region. These shifts can be attributed to the electron-withdrawing inductive effect (−I) of the bromine substituent, which decreases electron density on adjacent aromatic carbons and leads to slight deshielding. The overall pattern remains consistent with a highly conjugated and electron-deficient aromatic system. The preservation of the aromatic carbon framework and the characteristic imide carbonyl resonance in both compounds confirm the successful synthesis of the targeted conjugates.

The formation of the quaternized species was clearly evidenced by the presence of characteristic aliphatic methylene signals at ~5.6–5.7 ppm (4H) in the ^1^H NMR spectra, attributable to the –CH_2_–CH_2_– bridge. Furthermore, the downward shifts observed in the aromatic region support the formation of a positively charged phenazinium center. FT-IR spectra confirmed that the imide functionality remained intact during alkylation by retaining the imide carbonyl absorptions (~1707–1711 cm^−1^). Mass spectrometric data were also consistent with the formation of the corresponding cationic species.

In general, spectroscopic data consistently confirm the successful formation of the target conjugates. The rational structural design combining an aromatic extension and controlled cationization provides a promising platform for investigating the DNA-binding affinity, photophysical properties, and potential bioactive applications of these DPPZ–naphthalimide systems.

### 2.2. DNA Binding Studies

#### 2.2.1. UV Titration

UV–Vis absorption spectroscopy is one of the most widely used and reliable techniques for investigating the interaction of small molecules with DNA [14,27,28]. This method enables sensitive monitoring of changes in the electronic transitions of aromatic chromophores upon association with nucleic acids. Because ligand binding alters the electronic environment and π-conjugation of the chromophore, measurable variations in absorption intensity and wavelength are commonly observed. The nature of these spectral changes provides valuable insight into the binding mode. Classical intercalative binding between DNA base pairs typically results in pronounced hypochromism accompanied by a bathochromic (red) shift, attributed to strong π–π stacking interactions and partial orbital overlap between the ligand and the base pairs [5,29]. In contrast, groove binding or external electrostatic association generally produces smaller changes in intensity with minimal or no wavelength shift. For G4 structures, ligands frequently interact through end-stacking on the terminal G-tetrads, leading to moderate hypochromism and subtle bathochromic shifts due to π-surface stacking rather than deep helix insertion. Therefore, UV–Vis spectral analysis serves as a valuable preliminary tool for distinguishing interaction modes and estimating binding affinity. To quantitatively compare the ds-DNA and G4 DNA-binding affinities of the compounds, the intrinsic binding constants *K*_b_ were obtained according to the following Equation (1):

[DNA]/(*ε*_A_ − *ε*_f_) = [DNA]/(*ε*_B_ − *ε*_f_) + 1/*K*_b_ (*ε*_B_ − *ε*_f_) (1)

where [DNA] is in base pairs, *ε*_A_ is the absorption coefficient of the compound for each measurement, and *ε*_f_ and *ε*_B_ are the absorptivity values of the free and the fully bound forms of the compound.

For compound **1**, gradual addition of G4 and ds-DNA results in a clear hyperchromic effect, with only minimal bathochromic shift (Figure S15).

The hyperchromic shift suggests a more external or surface-associated interaction. In the case of G4 DNA, this behavior is consistent with partial end-stacking on the terminal G-tetrads or a conformational reorganization of the ligand upon binding, rather than deep insertion into the DNA structure. The binding constants (*K*_b_ = 0.9 × 10^8^ M^−1^ for G4 vs. 1.38 × 10^5^ M^−1^ for ds-DNA) indicate clear quadruplex preference (~10^3^ selectivity), despite the absence of strong hypochromism.

**Figure 2:**
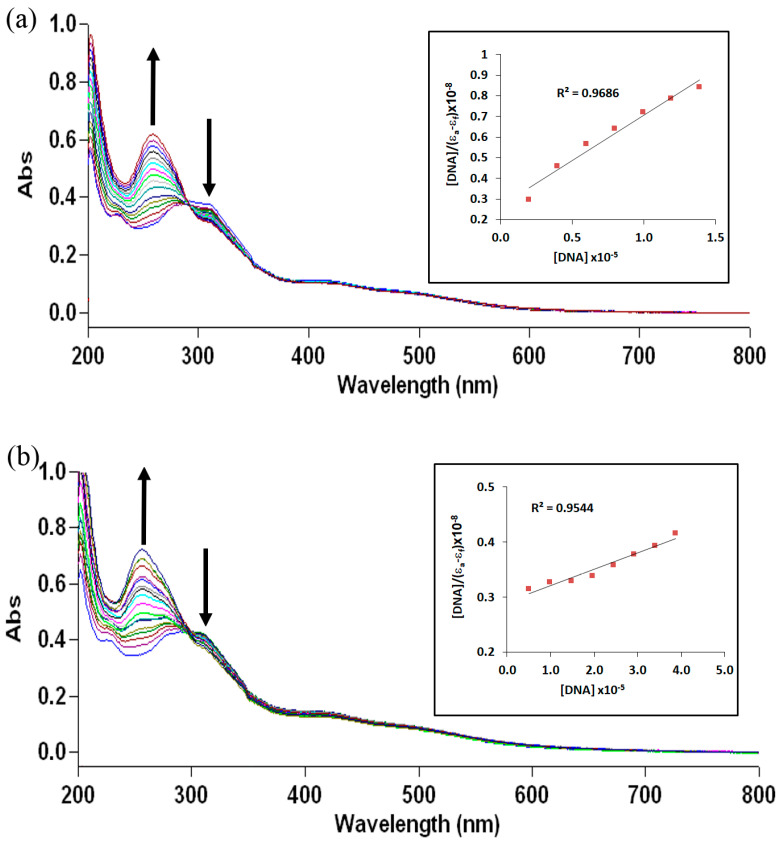
UV–Vis spectra of compound **1-q** with increasing amounts of ds-DNA (**a**) and G4-DNA (**b**). Arrows indicate the direction of spectral changes upon gradual addition of DNA.

Upon quaternization, compound **1-q** (Figure 2a,b) displays a different spectral profile. Although the dominant feature remains intensity variation rather than pronounced redshift, the binding constant toward G4 increases dramatically (7.4 × 10^8^ M^−1^), while duplex affinity decreases (0.3 × 10^5^ M^−1^).

The introduction of a permanent positive charge likely enhances electrostatic pre-organization near the phosphate backbone, enabling more effective π-surface stacking onto the G-tetrads. The reduced duplex affinity, on the other hand, suggests that steric or conformational constraints limit efficient intercalation into the B-DNA structure.

A different trend is observed for the brominated neutral analog compound **2** (Figure S16). Compared to **1**, **2** shows increased binding affinity toward both G4 (1.77 × 10^8^ M^−1^) and ds-DNA (2.5 × 10^5^ M^−1^).

The bromine substituent likely enhances ligand polarizability and dispersion-driven π–π interactions. Halogen atoms are known to increase noncovalent stabilization in biomolecular systems through enhanced van der Waals contacts and possible σ-hole interactions [30,31].

**Figure 3:**
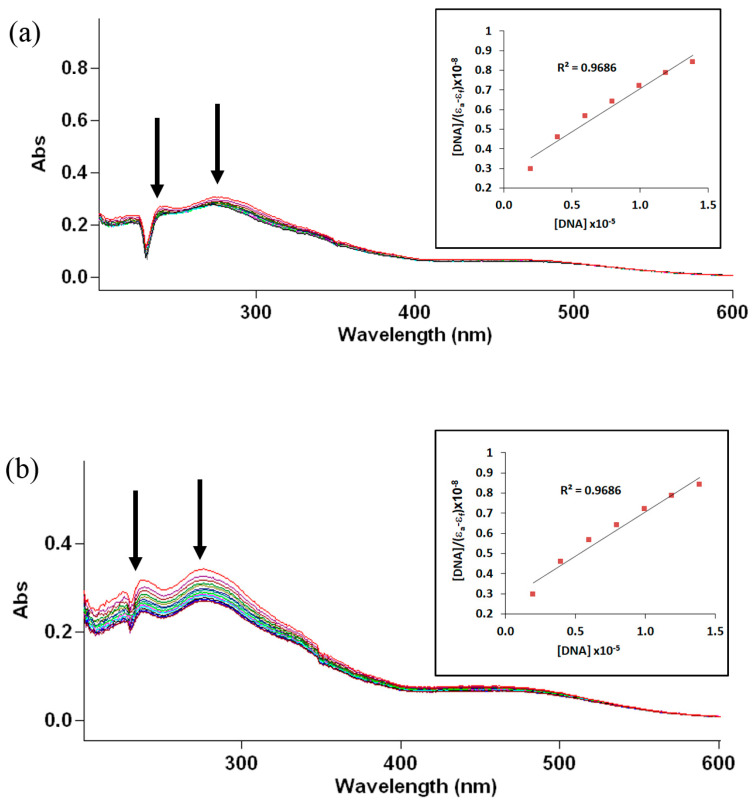
UV–Vis spectra of compound **2-q** with increasing amounts of ds-DNA (**a**) and G4-DNA (**b**). Arrows indicate the direction of spectral changes upon gradual addition of DNA.

Finally, the quaternized brominated derivative **2-q** (Figure 3a,b) exhibits the strongest overall binding in the series, with *K*_b_ values of 13.2 × 10^8^ M^−1^ for G4 and 13.8 × 10^5^ M^−1^ for ds-DNA.

In this case, the combined effects of extended π-conjugation, bromine-induced polarizability, and permanent cationic charge act synergistically to enhance DNA association. Unlike **1-q**, where quaternization selectively amplifies G4 binding, **2-q** shows a global increase in affinity toward both DNA topologies. This suggests that in the brominated scaffold, electrostatic attraction and enhanced dispersive stacking collectively stabilize both quadruplex end-stacking and duplex interactions.

In general, UV–Vis data indicate that DNA recognition in this ligand class is achieved through a delicate interplay between π-conjugation, the electronic effects of substituents, and cationization. The DPPZ–anhydride framework provides the primary stacking platform, bromine substitution enhances polarizability-driven stabilization, and quaternization modulates electrostatic contributions in a structure-dependent manner. While all compounds behave as strong G4 binders (10^8^–10^9^ M^−1^ range) [10,14,15,32,33,34,35], maximal topology selectivity is achieved with **1-q**, whereas maximal overall affinity is observed for **2-q**. These findings underscore the importance of fine structural tuning in the design of selective quadruplex-targeting ligands.

#### 2.2.2. FID Studies

The Thiazole Orange (TO) molecule, which emits a very weak signal in aqueous solutions, exhibits high fluorescence upon binding to G4 or double-stranded DNA. In this displacement method, the initial emission of the TO-DNA mixture is recorded, and the subsequent decrease in fluorescence is monitored as increasing amounts of ligand are added [36]. If the ligand binds more strongly than TO, it displaces it from the DNA, causing the emission to drop. For each molecule, the ligand concentration required to reduce the TO emission by 50% (DC_50_) was calculated. These results were then used as a standard to compare the G4-DNA selectivity of the different compounds.

For compound **1**, the FID profile indicates a moderate decrease in fluorescence intensity in both ds-DNA and G4 systems (Figure S17).

**Figure 4:**
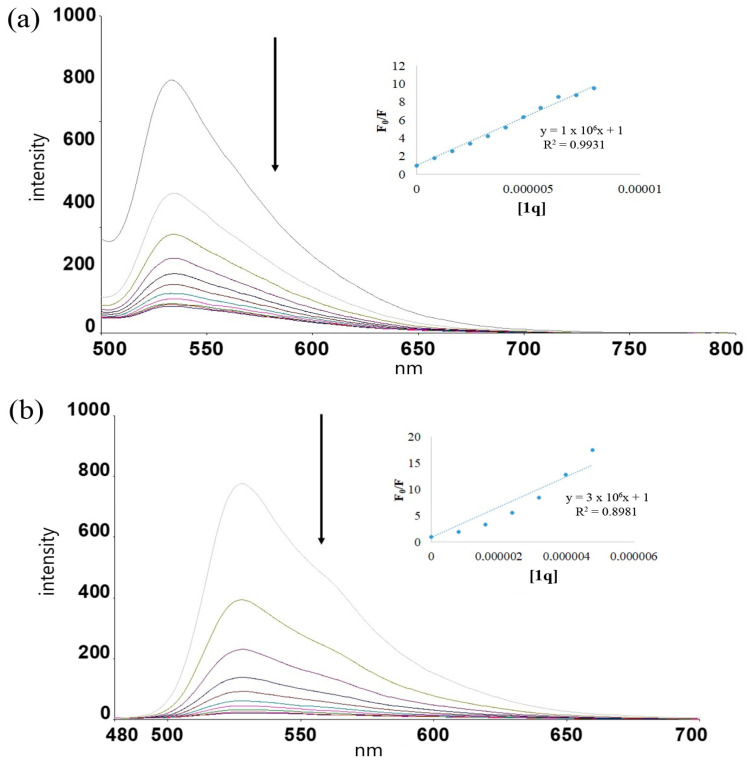
Emission spectrum obtained by adding increasing amounts of **1-q** onto the TO-dsDNA mixture (**a**) and the TO-G4 DNA mixture (**b**). Arrows indicate the direction of spectral changes upon gradual addition of **1-q**.

In contrast, **1-q** shows a markedly stronger fluorescence quenching effect, particularly in the G4 system (Figure 4).

The increased planarity and extended π-conjugation introduced by the DPPZ–anhydride framework likely promote stronger π–π stacking interactions with terminal G-tetrads, explaining the improved G4 stabilization and displacement efficiency.

Compound **2** demonstrates a binding profile similar in trend but slightly weaker than its quaternized analog (Figure S18).

**Figure 5:**
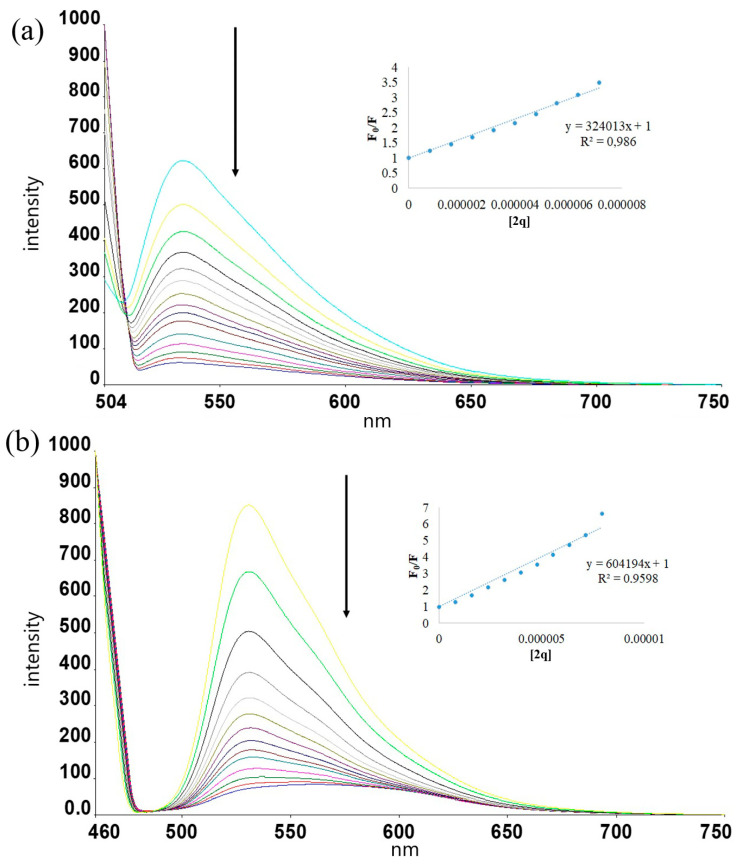
Emission spectrum obtained by adding increasing amounts of **2-q** onto the TO-dsDNA mixture (**a**) and the TO-G4 DNA mixture (**b**). Arrows indicate the direction of spectral changes upon gradual addition of **2-q**.

The most striking results are observed for **2-q**. This strong displacement of TO from G4-DNA (Figure 5) is fully consistent with its high binding constant as obtained from UV–Vis titration.

DC_50_ values were determined from three independent experiments and are reported as mean ± standard deviation (SD). The relative standard deviation (%RSD) values were below 5% for all compounds, indicating good reproducibility and reliability of the FID measurements (Tables S1 and S2). For the neutral compounds, moderate displacement efficiencies were observed. Compound **1** exhibited DC_50_ values of 12.5 µM (ds-DNA) and 23.2 µM (G4-DNA), indicating slightly stronger affinity for duplex DNA but overall moderate binding strength. Similarly, compound **2** showed even weaker binding, with DC_50_ values of 22.9 µM (ds-DNA) and 39.5 µM (G4-DNA). The higher DC_50_ values of **2** relative to **1** suggest that bromine substitution alone does not enhance DNA affinity; on the contrary, it appears to slightly reduce binding efficiency in the absence of quaternization. This may be attributed to the electron-withdrawing character of Br, which can subtly decrease electron density over the aromatic system and attenuate π–π stacking interactions.

In sharp contrast, the quaternized derivatives displayed dramatically enhanced displacement efficiency. Compound **1-q** showed DC_50_ values of 1.0 µM (ds-DNA) and 0.33 µM (G4-DNA), representing approximately a 12-fold enhancement toward ds-DNA and nearly a 70-fold enhancement toward G4-DNA compared to compound **1**. Notably, the submicromolar DC_50_ value for G4-DNA clearly indicates strong and preferential G4 binding.

Compound **2-q** also demonstrated significantly improved affinity relative to its neutral analog, with DC_50_ values of 1.66 µM (ds-DNA) and 3.08 µM (G4-DNA). Although **2-q** binds more strongly than **2** in both DNA forms, its G4 selectivity is less pronounced compared to **1-q**. This suggests that while quaternization is the dominant factor enhancing affinity, the bromine substituent subtly modulates G4 recognition efficiency within the cationic framework.

The strong correlation between low DC_50_ values (FID) and high intrinsic binding constants (*K*_b_) obtained from UV–Vis titrations further validates the binding model proposed for this series. DPPZ–anhydride conjugation enhances planarity and π-surface area, promoting stacking interactions, while quaternization dramatically strengthens electrostatic stabilization. Bromine substitution, in contrast, plays a secondary and fine-tuning electronic role.

Overall, these results demonstrate that π-extension and cationic character are the primary determinants of high-affinity and G4-selective DNA recognition in this ligand family, with compound **1-q** emerging as the most promising G4-targeting candidate.

Compound **1-q** exhibits submicromolar G4 displacement efficiency (DC_50_ = 0.33 µM), placing it within the potency range of established G4 ligands such as BRACO-19 [33] and pyridostatin. Although its selectivity ratio (~3-fold) is lower than that of telomestatin-class ligands [32], its binding strength clearly surpasses many classical intercalators, identifying it as a promising G4-targeting scaffold.

#### 2.2.3. Competition Dialysis Studies

Competitive dialysis is a widely employed and reliable method for the quantitative evaluation of structure-selective DNA-binding compounds [37,38]. This technique enables simultaneous comparison of ligand affinity toward different DNA conformations under identical equilibrium conditions. In a typical competition dialysis assay, dialysis units containing defined DNA structures are immersed in a common external solution of the test compound. Following equilibration, the ligand distributes among the compartments according to its relative binding affinities for each DNA structure.

After equilibrium is reached, the concentration of ligand bound within each dialysis unit is determined spectroscopically. The apparent binding constant (*K*_app_) is then calculated according to Equation (2):

*K*_app_ = *C_b_*/[*C_f_* × ([DNA]_total_ − *C_b_*)] *C_f_* (2)

where *C_b_* and *C_f_* are the bound and free ligand concentrations, respectively (C*_f_* = 5 μM), [DNA]_total_ is the total DNA concentration (200 μM).

The resulting *K*_app_ values provide a direct measure of relative affinity and allow quantitative comparison of ligand selectivity between duplex and G4-DNA structures.

Competitive dialysis experiments provided an independent and equilibrium-based assessment of DNA binding preference across the ligand series. The obtained binding values (relative affinity constants) revealed a consistent preference for G4-DNA over ds-DNA in all compounds.

For compound **1**, the binding value toward ds-DNA was 5.3 × 10^3^, while G4-DNA binding reached 1.6 × 10^4^, corresponding to approximately 3-fold G4 preference. This moderate selectivity aligns well with FID results, where **1** showed weaker overall affinity but measurable G4 interaction.

Compound **1-q** exhibited ds-DNA binding of 2.4 × 10^3^ and G4-DNA binding of 1.1 × 10^4^, corresponding to roughly 4.6-fold G4 selectivity. Notably, while the absolute binding constant toward ds-DNA decreased relative to compound **1**, the quadruplex affinity remained comparatively high. This supports the notion that quaternization enhances structural discrimination rather than simply increasing nonspecific electrostatic binding.

For compound **2**, binding values were 8.1 × 10^3^ (ds) and 1.9 × 10^4^ (G4), yielding ~2.3-fold G4 preference. Although absolute affinity increased relative to compound **1**, selectivity was slightly reduced. This suggests that bromine substitution enhances overall DNA affinity but does not proportionally increase quadruplex discrimination.

The highest absolute binding values were observed for **2-q**, with 1.7 × 10^4^ (ds) and 3.5 × 10^4^ (G4). While this compound shows the strongest overall DNA binding in the series, its selectivity ratio (~2-fold) is lower than that of **1-q**. This indicates that the combined effects of bromine substitution and quaternization maximize affinity but partially reduce structural selectivity.

### 2.3. BSA Binding Studies

**Figure 6:**
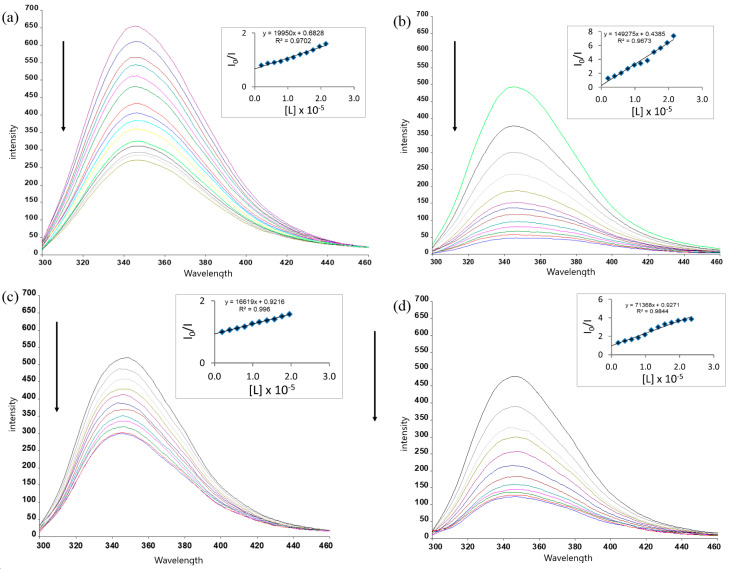
Fluorescence emission spectra of BSA in the presence of increasing concentrations of ligands and corresponding Stern–Volmer plots. (**a**) BSA with compound **1**, (**b**) BSA with **1-q**, (**c**) BSA with **2**, and (**d**) BSA with **2-q**. Progressive addition of the ligands results in a gradual decrease in the intrinsic fluorescence intensity of BSA, indicating effective quenching of the tryptophan residues. The inset graphs show the Stern–Volmer plots (I_0_/I vs. [L]) used to determine the quenching constants (*K*_sv_), revealing stronger quenching efficiency for the quaternized derivatives compared to the neutral ligands. Arrows indicate the direction of spectral changes upon gradual addition of **1**, **1-q**, **2** and **2-q**.

The interaction between the synthesized ligands and bovine serum albumin (BSA) was investigated by fluorescence quenching experiments. BSA exhibits a characteristic intrinsic fluorescence emission band around ~340–350 nm, mainly originating from the tryptophan residues. Upon gradual addition of the ligands, a systematic decrease in fluorescence intensity was observed without a significant shift in the emission maximum, indicating that the ligands interact with BSA and effectively quench its intrinsic fluorescence (Figure 6).

The quenching data were analyzed using the Stern–Volmer equation, and the corresponding Stern–Volmer quenching constants (*K*_sv_) were determined. The calculated *K*_sv_ values were 1.99 × 10^4^ M^−1^ for **1**, 1.49 × 10^5^ M^−1^ for **1-q**, 1.66 × 10^4^ M^−1^ for **2**, and 7.14 × 10^4^ M^−1^ for **2-q**. Similar binding strengths have been widely reported for various aromatic drugs and heteroaromatic ligands interacting with serum albumins. For example, the anticancer drug sorafenib exhibits a BSA binding constant of 6.8 × 10^4^ M^−1^, which falls within the same order of magnitude as the values observed in the present work [39]. More generally, many small organic drugs and metal–ligand complexes exhibit serum albumin binding constants in the 10^4^–10^6^ M^−1^ range, which is considered optimal for reversible drug–protein interactions that allow efficient transport in biological systems [40,41,42].

Among the investigated compounds, **1-q** displayed the highest *K*_sv_ value, indicating the strongest interaction with BSA. This enhancement can be attributed to the introduction of permanent positive charges through quaternization, which strengthens electrostatic interactions with negatively charged amino acid residues in the protein binding pockets. In contrast, bromine substitution had only a minor influence on the overall protein-binding strength, as evidenced by the similar *K*_sv_ values obtained for compounds **1** and **2**.

Overall, the results suggest that cationic modification plays a dominant role in promoting BSA binding, whereas halogen substitution mainly modulates the electronic properties of the ligand framework. The observed binding affinities fall within the typical range reported for small aromatic ligands interacting with serum albumins (10^4^–10^5^ M^−1^), indicating moderate and reversible protein binding, which is considered favorable for potential biological transport in serum.

## 3. Discussion

The combined UV–Vis, FID and competitive dialysis results reveal a consistent structure–activity relationship within this ligand series. The DPPZ–anhydride π-conjugated framework enables efficient DNA interaction, while quaternization markedly enhances binding strength through electrostatic stabilization.

UV–Vis titrations demonstrated significantly higher intrinsic binding constants for the quaternized derivatives, particularly toward G4-DNA. FID assays supported these findings, with submicromolar DC_50_ values—most notably for **1-q**—indicating efficient displacement of thiazole orange from G4 structures. Competitive dialysis experiments further confirmed preferential accumulation in G4 compartments under equilibrium conditions.

A distinction between affinity and selectivity is evident. Although **2-q** exhibits the highest overall binding constants, its increased duplex affinity reduces topology discrimination. In contrast, **1-q** combines strong binding with improved G4 selectivity across all three techniques, suggesting an optimal balance between π-stacking and electrostatic interactions. Bromine substitution appears to enhance global DNA affinity, likely through increased polarizability, but does not significantly improve quadruplex selectivity.

Overall, the data indicate that π-planarity, cationic character, and electronic modulation collectively govern DNA recognition, with **1-q** emerging as the most promising G4-targeting candidate in this series.

Fluorescence quenching experiments with BSA, the quaternized derivatives exhibited significantly higher quenching efficiencies, suggesting stronger interactions with the protein. This enhancement can be attributed to increased electrostatic attraction between the positively charged ligands and negatively charged residues in the BSA binding pockets. Overall, the observed binding strengths indicate moderate and reversible protein binding, which is generally considered favorable for the transport of small molecules in biological environments.

## 4. Materials and Methods

Commercially available reagents and solvents were purchased from Sigma-Aldrich (St. Louis, MO, USA) and used as received without further purification, with exceptions being explicitly mentioned. 1,8-naphthalic anhydride, 4-bromo-1,8-naphthalic anhydride, 1,2-dibromoethane, pyridine, and imidazole were used as received.

Solvents, including acetone, ethanol, and diethyl ether, were of analytical grade and used for purification and washing steps. Hydrochloric acid (HCl, 15%) was used for the precipitation of the target compounds.

All aqueous solutions were prepared using deionized water. Buffer solutions (PBS, Tris–HCl) used in spectroscopic studies were prepared according to standard procedures.

Calf thymus DNA (CT-DNA) purchased from Sigma-Aldrich (St. Louis, MO, USA) Sigma was prepared in solutions containing 100 mM KCl and 10 mM Tris (pH 7.5). These solutions exhibited a UV–Vis absorbance ratio of 1.8–1.9 at 260 and 280 nm (A_260_/A_280_ = 1.9), indicating minimal protein contamination in the DNA. The DNA concentration was determined in base pairs using spectrophotometry and a molar absorptivity value of 6600 M^−1^ cm^−1^ (260 nm).

The extinction coefficient of the DNA oligomer HTG21 (sequence: 5′-GGGTTAGGGTTAGGGTTAGGG-3′), purchased from Thermo Fisher Scientific (Waltham, MA, USA), was calculated using a nearest neighbor approximation based on mononucleotide. Double-distilled water was the solvent used for buffer preparation. Stock solutions of both CT-DNA and HTG21 were stored at 4 °C and utilized within a four-day timeframe.

Intramolecular G4 formation involved the following steps: Oligonucleotide samples, dissolved in various buffers, were heated to 90 °C for five minutes. Following gentle cooling to room temperature, the samples were incubated overnight at 4 °C. To enhance solubility, complex solutions were prepared by dissolving a weighed amount of the sample in 0.5 mL DMSO. These solutions were subsequently diluted (up to 150-fold without precipitation) with 100 mM KCl and 10 mM Tris (pH 7.5) to achieve the desired concentration.

### 4.1. Chemistry

#### 4.1.1. Synthesis of 2-(4-(Dipyrido[3,2-a:2′,3′-c]phenazin-11-yl)phenyl)-1H-benzo[de]isoquinoline-1,3(2H)-dione (**1**)

A mixture of amino-dppz (0.30 g, 1.01 mmol) and 1,8-Naphthalic anhydride (0.20 g, 1.01 mmol) was dissolved in pyridine (5.0 mL) and imidazole (5.0 g). The reaction mixture was refluxed at 120 °C under argon for 24 h. After cooling to room temperature, the reaction mixture was slowly poured into 55 mL of 15% HCl, affording a brownish precipitate, which was collected by filtration and washed with water, acetone and ethanol. Electrospray ionization mass spectrometry (MS): *m*/*z* = 478.45 [M + 1] (Figure S1). ^1^H-NMR (600 MHz, DMSO-*d*_6_ + TFA, *δ*, ppm): 10.08 (d, *J* = 8.3 Hz, 1H), 10.01 (d, *J* = 8.2 Hz, 1H), 9.36 (dd, *J* = 9.5, 4.8 Hz, 2H), 8.79 (d, *J* = 7.1 Hz, 2H), 8.63 (d, *J* = 9.0 Hz, 1H), 8.52 (s, 1H), 8.45 (d, *J* = 8.3 Hz, 2H), 8.28 (ddd, *J* = 20.6, 8.4, 5.3 Hz, 2H), 8.02 (d, *J* = 9.1 Hz, 1H), 7.93 (t, *J* = 7.8 Hz, 2H). (Figure S2). ^13^C NMR (600 MHz, DMSO-*d*_6_ + TFA, δ ppm): 121.16, 127.32, 127.69, 128.66, 129.59, 129.66, 130.28, 131.56, 131.89, 132.91, 133.52, 136.63, 138.10, 139.13, 139.51, 139.66, 143.08, 148.46, 165.95 (Figure S3). Fourier transform (FT) IR (*ν*, cm^−1^): 3106 (aromatic C–H), 1705 (C=O), 1668 (C=N), and no carbonyl peak belonging to anhydride was detected, as supposed (Figure S4).

#### 4.1.2. Synthesis of 2-(4-(Dipyrido[3,2-a:2′,3′-c]phenazin-11-yl)phenyl)-4-bromo-1H-benzo[de]isoquinoline-1,3(2H)-dione (**2**)

4-Bromo-1,8-naphthalic anhydride was used in place of 1,8-naphthalic anhydride, and the target product was prepared following the same procedure and reaction conditions employed for compound **1**. (MS): *m*/*z* = 544.1 [M–CH_2_] (Figure S5). ^1^H-NMR (600 MHz, DMSO-*d*_6_, *δ*, ppm): 9.61 (m, 1H), 9.56 (d, *J* = 8.1 Hz, 1H), 9.23 (m, 2H), 8.65 (m, 2H), 8.56 (m, 2H), 8.23 (s, 1H), 8.19 (m, 1H), 8.12 (dd, *J* = 9.6, 1.8 Hz, 1H), 8.03–7.97 (m, 3H). (Figure S6). ^13^C NMR (600 MHz, DMSO-*d*_6_, δ ppm): 123.08, 127.82, 128.54, 129.90, 130.46, 130.52, 131.12, 131.16, 131.39, 132.02, 133.04, 135.30, 136.22, 138.13, 138.38, 141.42, 142.98, 143.35, 143.42, 143.46, 150.18, 164.27 (Figure S7). Fourier transform (FT) IR (*ν*, cm^−1^): 3106 (aromatic C–H), 1705 (C=O), 1668 (C=N), and no carbonyl peak belonging to anhydride was detected, as supposed (Figure S8).

#### 4.1.3. Synthesis of Ethane-1,2-diyl-bridged Dipyrido[3,2-a:2′,3′-c]phenazinium–naphthalimide dibromide (**1-q**)

100 mg (0.21 mmol) of compound **1** was mixed with 10 mL of 1,2-dibromoethane, and the mixture was stirred at 130 °C for 12 h under reflux. The yellow precipitate formed as a result of the reaction was filtered. It was washed with acetone and ether and dried. (MS): *m*/*z* = 505.1 [M + 1] (Figure S9). ^1^H-NMR (600 MHz, DMSO-*d*_6_, *δ*, ppm): 10.44 (d, *J* = 8.2 Hz, 1H), 10.39 (d, *J* = 8.3 Hz, 1H), 9.86 (dd, *J* = 8.4, 5.7 Hz, 2H), 8.89 (ddd, *J* = 14.3, 8.5, 5.7 Hz, 2H), 8.78–8.72 (m, 2H), 8.60–8.53 (m, 4H), 8.38 (dd, *J* = 8.8, 2.1 Hz, 1H), 7.95 (t, *J* = 7.7 Hz, 2H), 5.69 (s, 4H) (Figure S10). Fourier transform (FT) IR (*ν*, cm^−1^): 3015 (aromatic C–H), 2900 (aliphatic C-H), 1711 (C=O), 1668 (C=N) (Figure S11).

#### 4.1.4. Synthesis of Ethane-1,2-diyl-bridged Dipyrido[3,2-a:2′,3′-c]phenazinium–bromonaphthalimide dibromide (**2-q**)

100 mg (0.18 mmol) of compound **2** was mixed with 10 mL of 1,2-dibromoethane, and the mixture was stirred at 130 °C for 12 h under reflux. The yellow precipitate formed as a result of the reaction was filtered. It was washed with acetone and ether and dried. (MS): *m*/*z* = 584.1 [M+] (Figure S12). ^1^H-NMR (600 MHz, DMSO-*d*_6_, *δ*, ppm): ^1^H NMR (600 MHz) δ 10.28 (d, *J* = 8.5 Hz, 1H), 10.16 (d, *J* = 8.6 Hz, 1H), 9.75 (d, *J* = 5.8 Hz, 1H), 9.66 (d, *J* = 5.8 Hz, 1H), 9.23 (q, *J* = 10.8, 8.4 Hz, 2H), 8.74 (ddd, *J* = 19.5, 8.4, 5.6 Hz, 2H), 8.27 − 7.92 (m, 6H), 5.61 (s, 4H) (Figure S13). Fourier transform (FT) IR (*ν*, cm^−1^): 3058 (aromatic C–H), 2900 (aliphatic C-H), 1707 (C=O), 1667 (C=N) (Figure S14).

### 4.2. DNA Studies

#### 4.2.1. Absorption Titrations

Stock solutions of the synthesized compounds were initially prepared in spectroscopic-grade DMSO to ensure complete dissolution. These stock solutions were subsequently diluted with Tris buffer (10 mM Tris–HCl, 100 mM KCl, pH 7.5) to obtain a final compound concentration of 20 μM. The final DMSO content in the measurement solutions was kept below 1% (*v*/*v*) to avoid interference with DNA structure.

Calf thymus DNA (CT-DNA) stock solution (1.25 mM in nucleotide phosphate, n.t.) was prepared in the same buffer and stored at 4 °C. The concentration of CT-DNA was determined spectrophotometrically using an extinction coefficient of *ε*_260_ = 6600 M^−1^cm^−1^. The HTG21 oligonucleotide was dissolved in buffer, heated to 95 °C for 5 min, and slowly cooled to room temperature to allow proper G4 formation prior to use. The final concentration of HTG21 in titration experiments was 1 μM.

UV–Vis absorption titrations were performed using a 10 mm path-length quartz cuvette equipped with a stopper to prevent evaporation. A fixed concentration of compound (20 μM) was titrated by incremental additions of CT-DNA or HTG21 solution. After each addition, the mixture was gently mixed and incubated for 10 min at room temperature to allow equilibration before spectral acquisition.

Absorption spectra were recorded over the range 200–600 nm. Baseline corrections were applied using buffer solutions as a reference. Dilution effects were corrected mathematically where necessary.

#### 4.2.2. Fluorescent Intercalator Displacement (FID) Assays

Competitive binding experiments were performed using thiazole orange (TO) as a fluorescent probe.

CT-DNA (15 μM in base pairs) or HTG21 (1 μM) was pre-incubated with TO at room temperature for 15 min to allow formation of the DNA–TO complex. The excitation wavelength was set at 467 nm, and emission spectra were recorded between 480 and 700 nm.

The DNA–TO complex was titrated with incremental additions of the synthesized compounds. After each addition, the mixture was gently mixed and incubated for 5–10 min prior to fluorescence measurement to ensure equilibrium.

Fluorescence intensity changes were monitored at the emission maximum of TO. The decrease in fluorescence intensity was used to evaluate the displacement efficiency of each compound. All measurements were performed at room temperature under identical instrumental settings to ensure comparability.

#### 4.2.3. Equilibrium Dialysis Experiments

Equilibrium dialysis experiments were conducted to quantitatively assess DNA-binding affinity.

Mini dialysis tubes with a molecular weight cut-off (MWCO) of 3500 Da (Slide-A-Lyzer MINI) were used. Dialysis was performed in 20 mM Tris–HCl buffer (pH 7.4) containing 20 mM NaCl. Sodium chloride was selected to avoid possible competitive interactions of potassium ions with surfactants during subsequent SDS treatment.

Dialysis chambers were filled with DNA solution (200 μM in nucleotide phosphate) or buffer only (control). The external solution contained the compounds at a defined concentration. The systems were incubated at 25 °C for 24 h under gentle agitation to allow equilibrium to be reached.

After incubation, aliquots were withdrawn and mixed with 10% (w/v) SDS stock solution to obtain a final SDS concentration of 1% (w/v), facilitating dissociation of DNA–compound complexes. Samples were centrifuged to remove any precipitates, and the concentration of DNA-bound compound was determined spectrophotometrically using the characteristic absorption band of the compounds.

### 4.3. BSA Studies

The interaction between the synthesized ligands and bovine serum albumin (BSA) was investigated using fluorescence spectroscopy. A stock solution of BSA was prepared in phosphate-buffered saline (PBS, pH 7.4) and diluted to obtain a final protein concentration of 4 μM. Fluorescence measurements were carried out by titrating the BSA solution with aliquots of the ligand stock solution.

Ligand solutions (1 mM) were prepared in a minimal amount of DMSO and subsequently diluted with PBS buffer. During the titration experiment, 10 μL portions of the ligand solution were successively added to the BSA solution, and the fluorescence spectra were recorded after each addition. The samples were allowed to equilibrate briefly before measurement.

The intrinsic fluorescence emission spectra of BSA were recorded in the range of 300–500 nm upon excitation at 280 nm. The decrease in fluorescence intensity of BSA with increasing ligand concentration was analyzed using the Stern–Volmer equation to determine the quenching constants (*K*_sv_).

All measurements were performed at room temperature, and the corresponding Stern–Volmer plots (I_0_/I vs. [L]) were constructed to evaluate the quenching efficiency of each ligand.

## 5. Conclusions

The combined spectroscopic and biochemical analyses highlight the critical roles of π-planarity, cationic character, and electronic modulation in governing DNA recognition within this ligand series. Quaternization markedly enhances binding affinity, particularly toward G4- DNA, as evidenced by UV–Vis titrations, FID assays, and competitive dialysis experiments. Among the derivatives, 1-q demonstrates the optimal balance between strong binding and selective G4 recognition, whereas bromine substitution increases overall DNA affinity without improving quadruplex selectivity. Fluorescence quenching studies with BSA indicate moderate, reversible protein interactions, supporting potential biological transport. Collectively, these findings identify **1-q** as the most promising candidate for selective G4 targeting, providing valuable insights for the rational design of DNA-interacting ligands.

## Data Availability

The original contributions presented in this study are included in the article/Supplementary Materials. Further inquiries can be directed to the corresponding author.

## Supplementary Materials

The following supporting information can be downloaded at: https://www.mdpi.com/article/10.3390/ph19040575/s1, Figure S1. ESI-MS spectrum of **1**. Figure S2. 1H NMR spectrum of **1**. Figure S3. 13C NMR spectrum of **1**. Figure S4. FT-IR spectrum of **1**. Figure S5. ESI-MS spectrum of **2**. Figure S6. 1H NMR spectrum of **2**. Figure S7. 13C NMR spectrum of **2**. Figure S8. FT-IR spectrum of **2**. Figure S9. ESI-MS spectrum of **1-q**. Figure S10. 1H NMR spectrum of **1-q**. Figure S11. FT-IR spectrum of **1-q**. Figure S12. ESI-MS spectrum of **2-q**. Figure S13. 1H NMR spectrum of **2-q**. Figure S14. FT-IR spectrum of **2-q**. Figure S15. UV–Vis spectra of compound **1** with increasing amounts of ds-DNA (a) and G4-DNA (b). Figure S16. UV–Vis spectra of compound **2** with increasing amounts of ds-DNA (a) and G4-DNA (b). Figure S17. Emission spectrum obtained by adding increasing amounts of **1** onto the TO-dsDNA mixture (a) and the TO-G4 DNA mixture (b). Figure S18. Emission spectrum obtained by adding increasing amounts of **2** onto the TO-dsDNA mixture (a) and the TO-G4 DNA mixture (b). Table S1. DC_50_ values (µM) for ligand binding to double-stranded DNA (dsDNA) determined by FID assay (mean ± SD, n = 3). Table S2. DC_50_ values (µM) for ligand binding to G-quadruplex DNA determined by FID assay (mean ± SD, n = 3).

## Abbreviations

BSA	Bovine Serum Albumin
CT-DNA	Calf Thymus DNA
DC_50_	Displacement Concentration at 50%
ds-DNA	Double-Stranded DNA
EB	Ethidium Bromide
FID	Fluorescence Intercalator Displacement
G4	G-Quadruplex
*K*_b_	Intrinsic Binding Constant
*K*_app_	Apparent Binding Constant
TO	Thiazole Orange
UV–Vis	Ultraviolet–Visible Spectroscopy
