# Supplementary material for: DPPZ–Naphthalimide Conjugates as G-Quadruplex DNA Targeting Scaffolds: Design, Synthesis and Biomolecular Interaction Studies
Source: Pharmaceuticals (Basel). 2026 Apr 2;19(4):575. doi: 10.3390/ph19040575 (PMC13119045; doi:10.3390/ph19040575)
Supplement: Supplementary file 1 [file pharmaceuticals-19-00575-s001.zip › pharmaceuticals-4225277-supplementary.pdf]

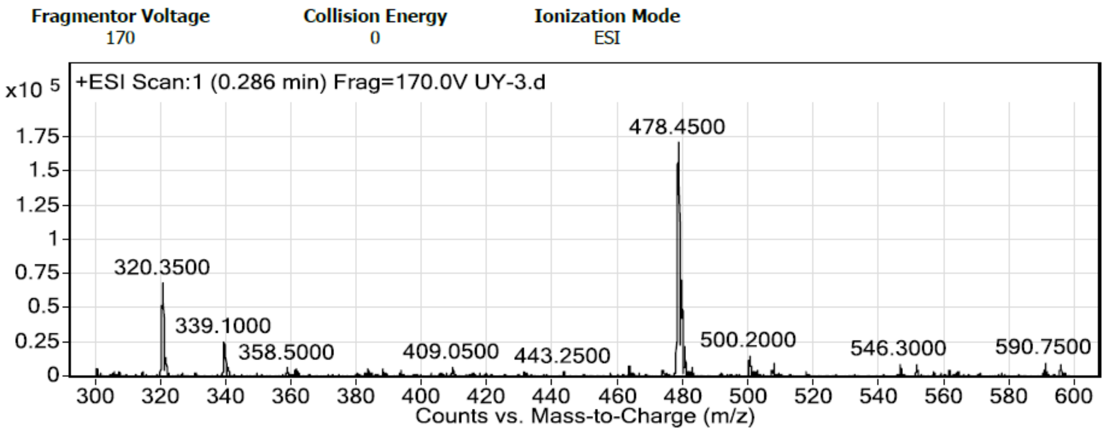

Figure S1. ESI-MS spectrum of 1.

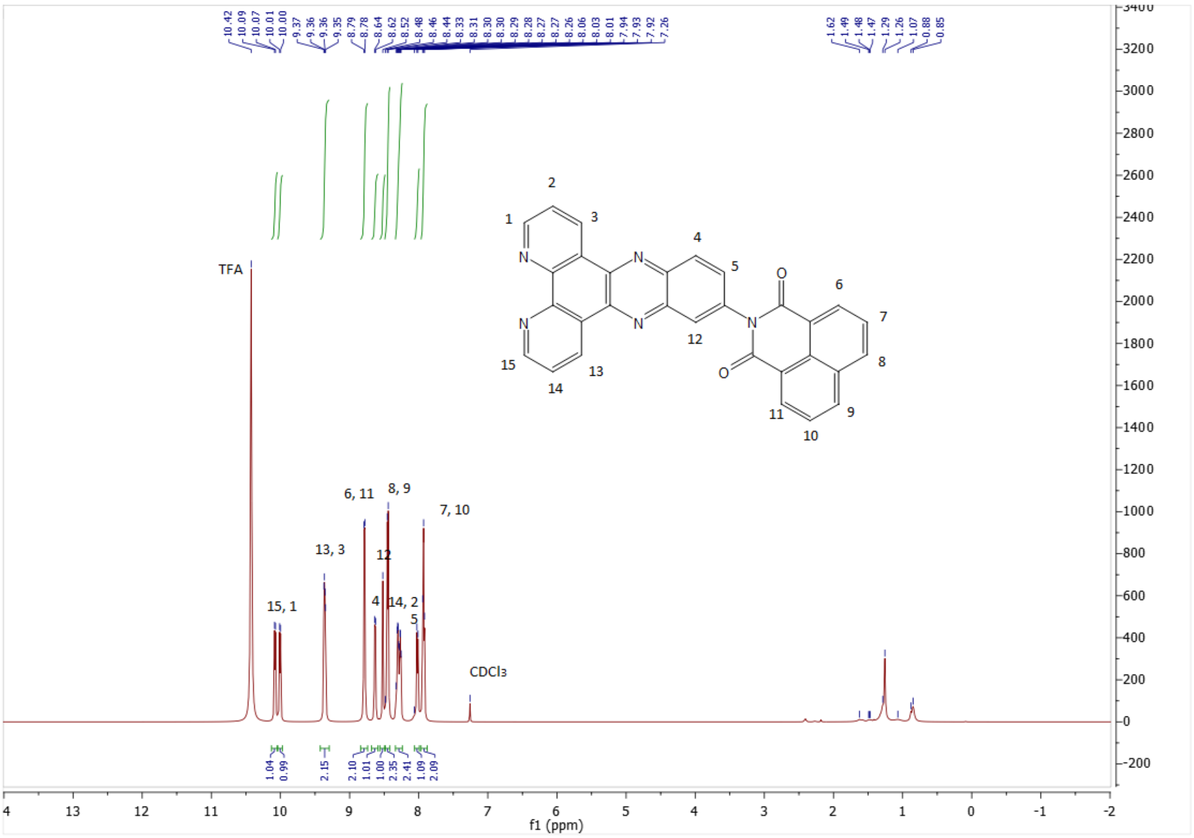

Figure S2. ^{1}H NMR spectrum of 1.

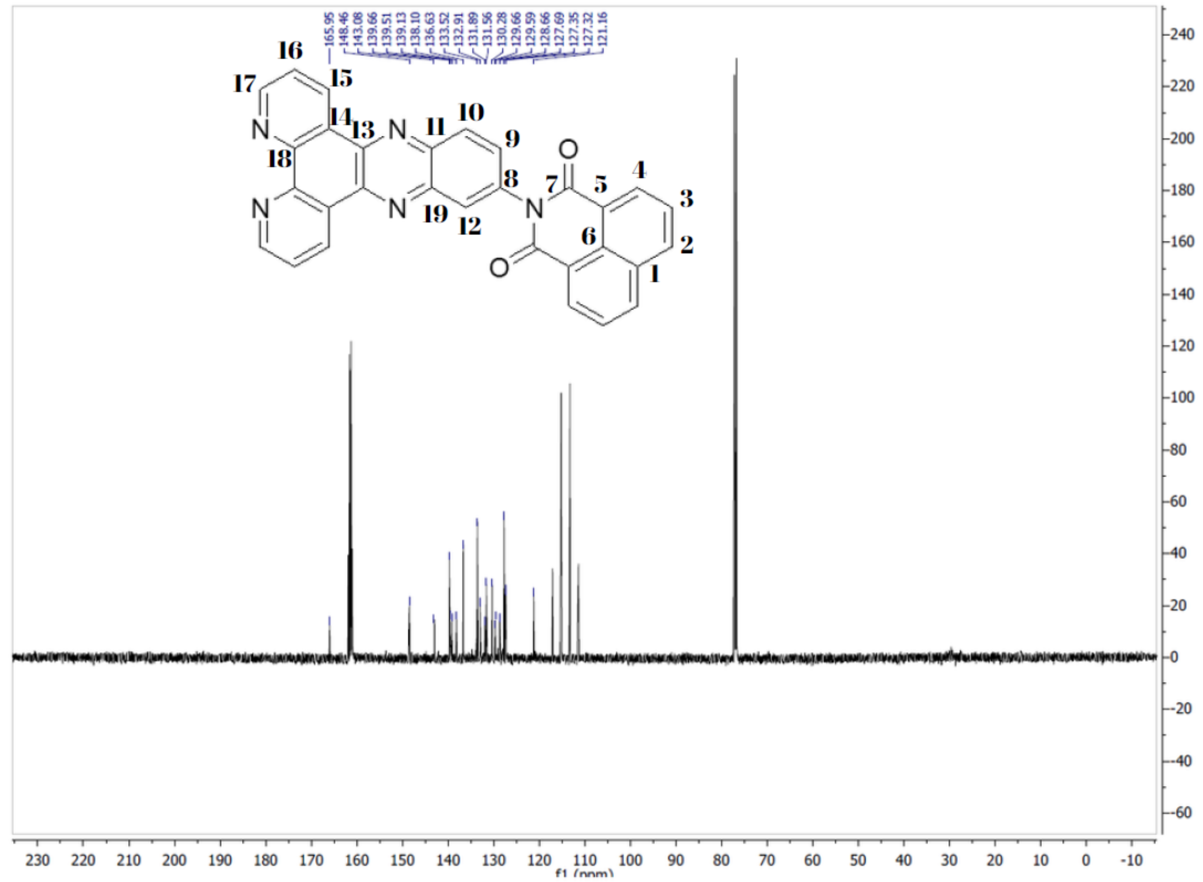

Figure S3. ^{13}C NMR spectrum of 1.

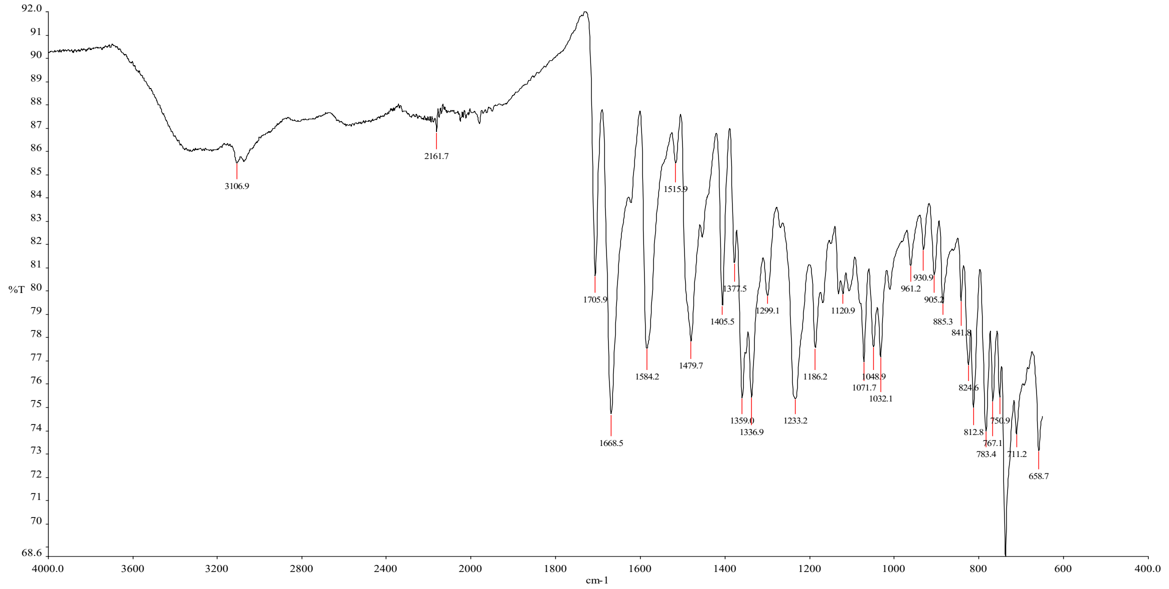

Figure S4. FT-IR spectrum of 1.

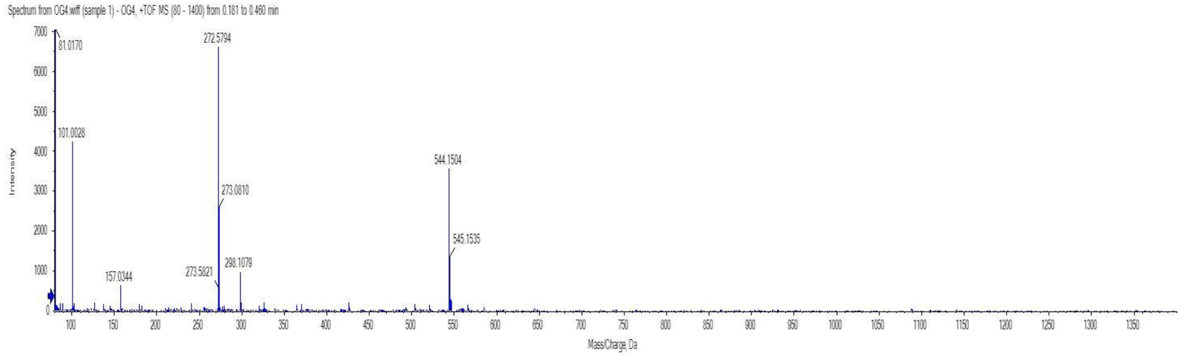

Figure S5. ESI-MS spectrum of 2.

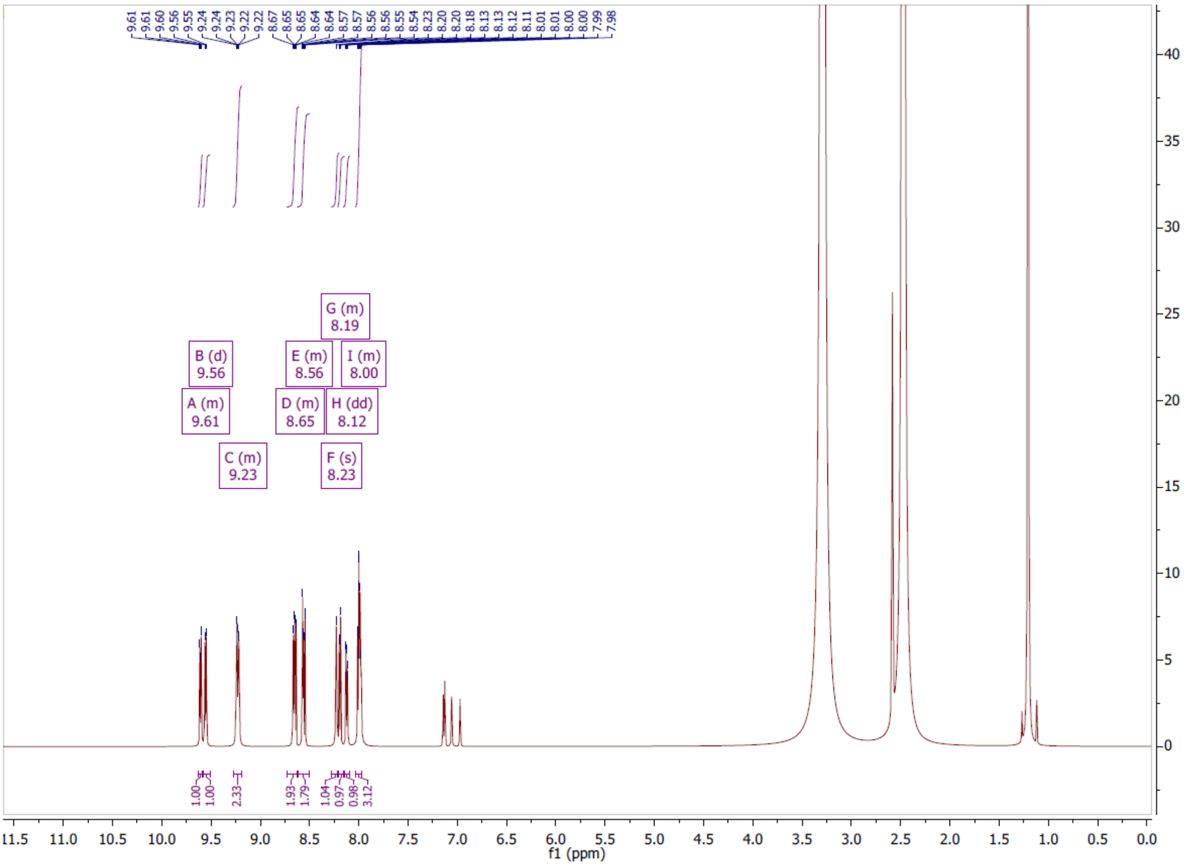

Figure S6. ^{1}H NMR spectrum of 2.

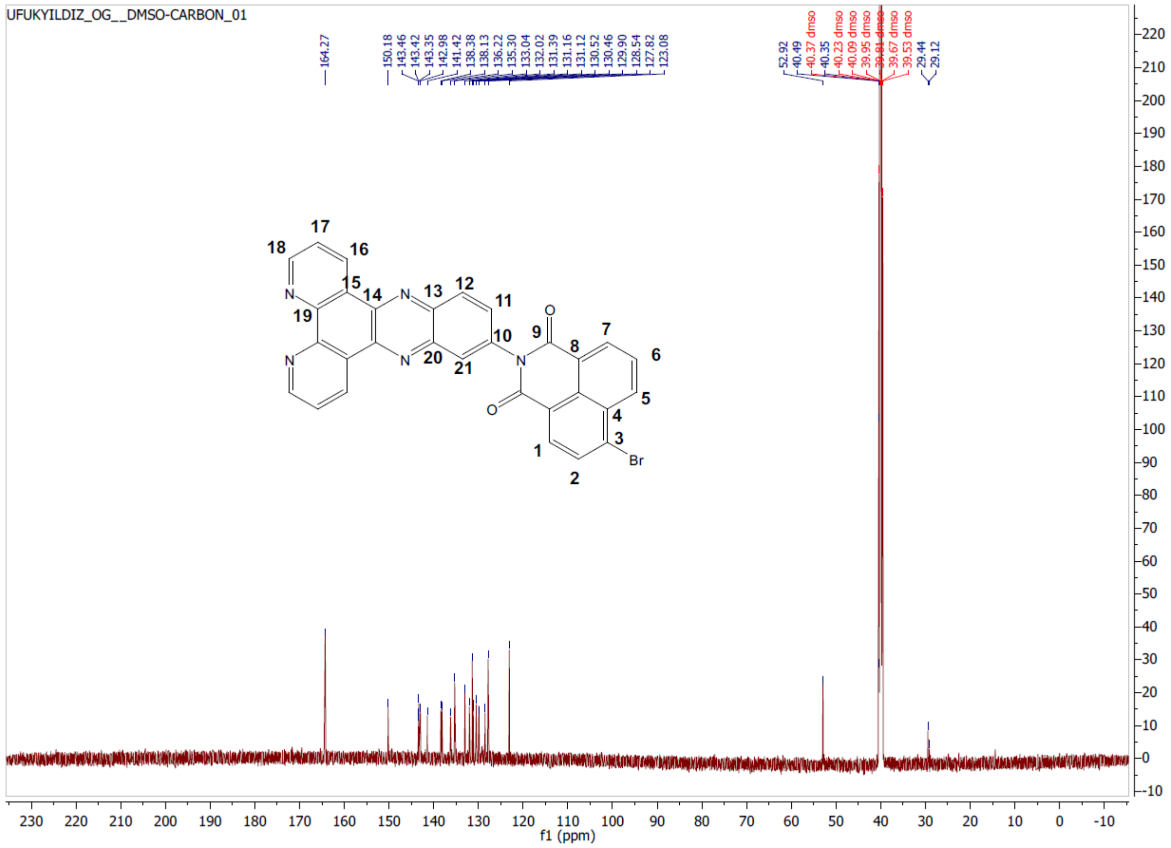

Figure S7. ^{13}C NMR spectrum of 2.

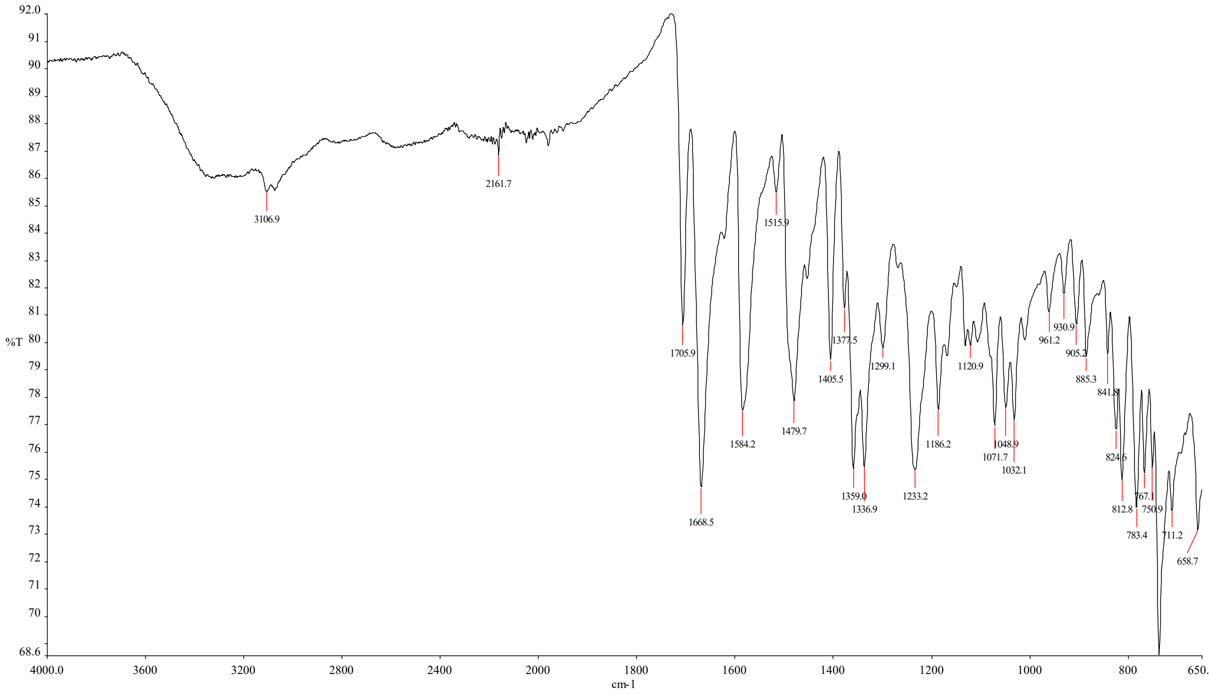

Figure S8. FT-IR spectrum of 2.

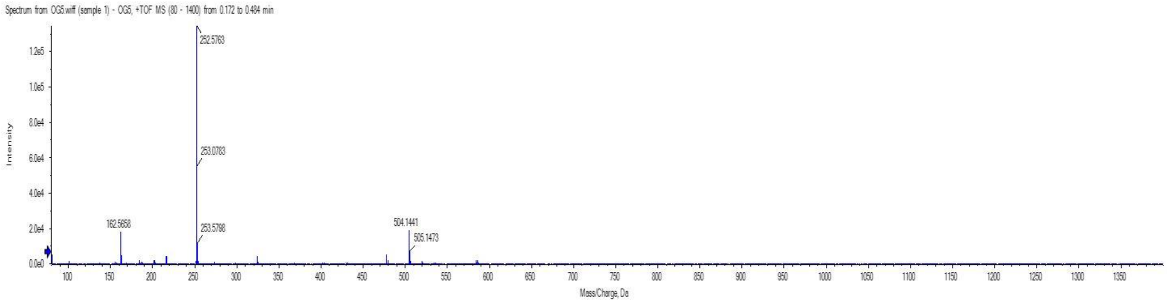

Figure S9. ESI-MS spectrum of 1-q.

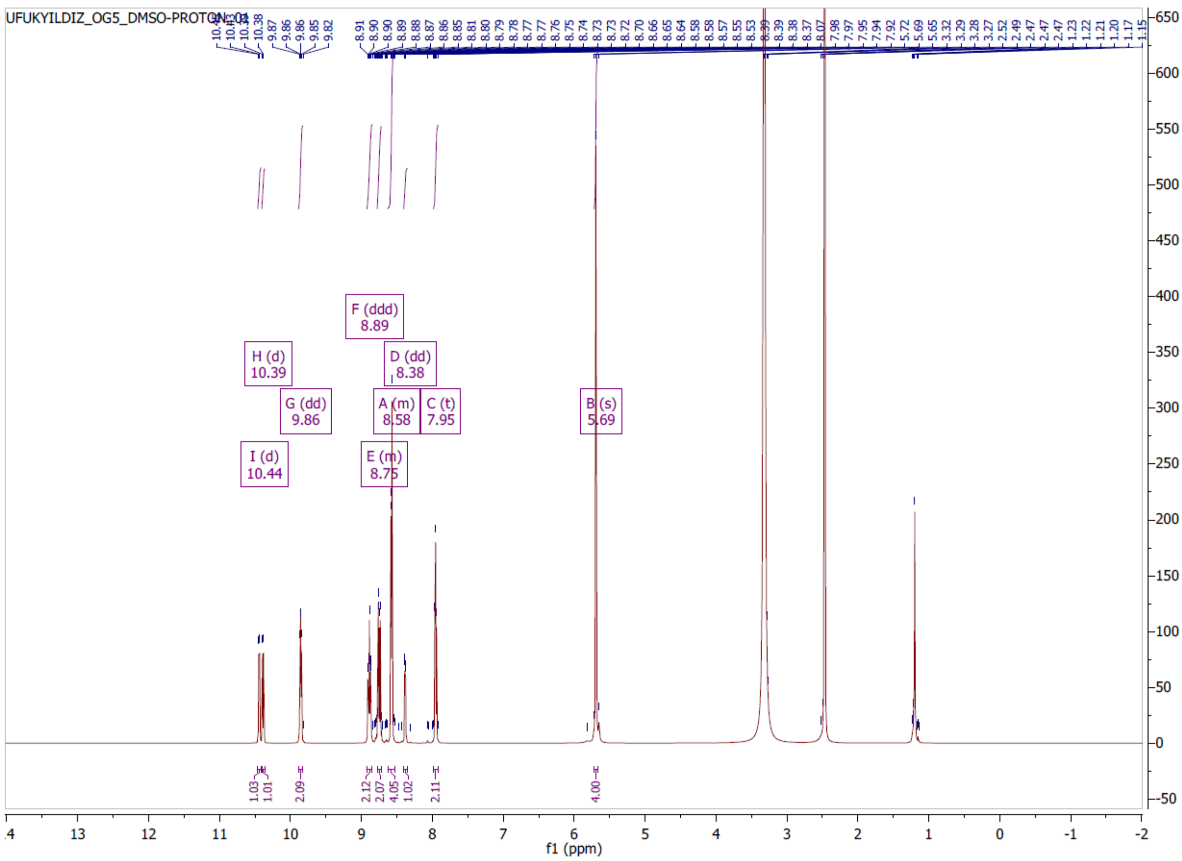

Figure S10. ^{1}H NMR spectrum of 1-q.

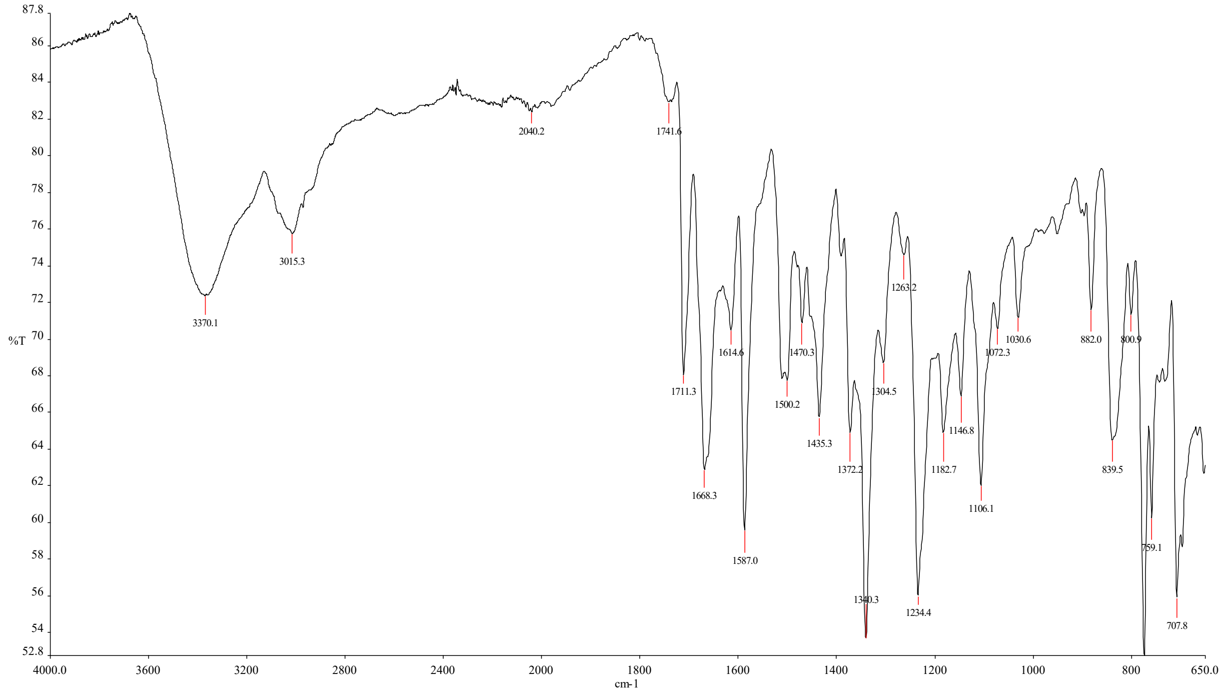

Figure S11. FT-IR spectrum of 1-q.

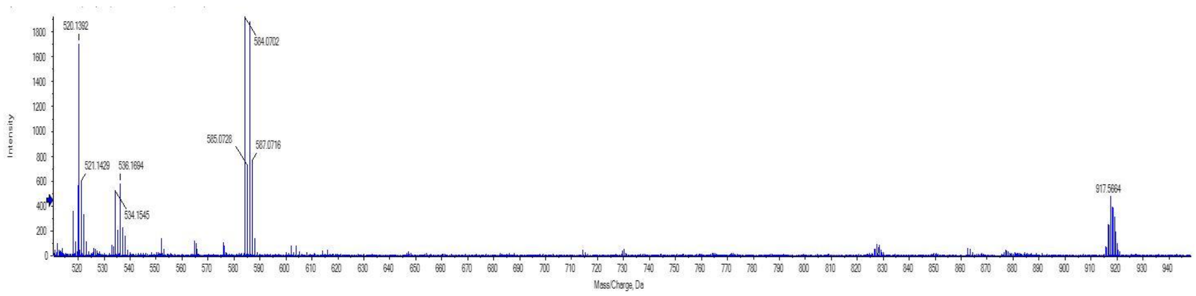

Figure S12. ESI-MS spectrum of 2-q.

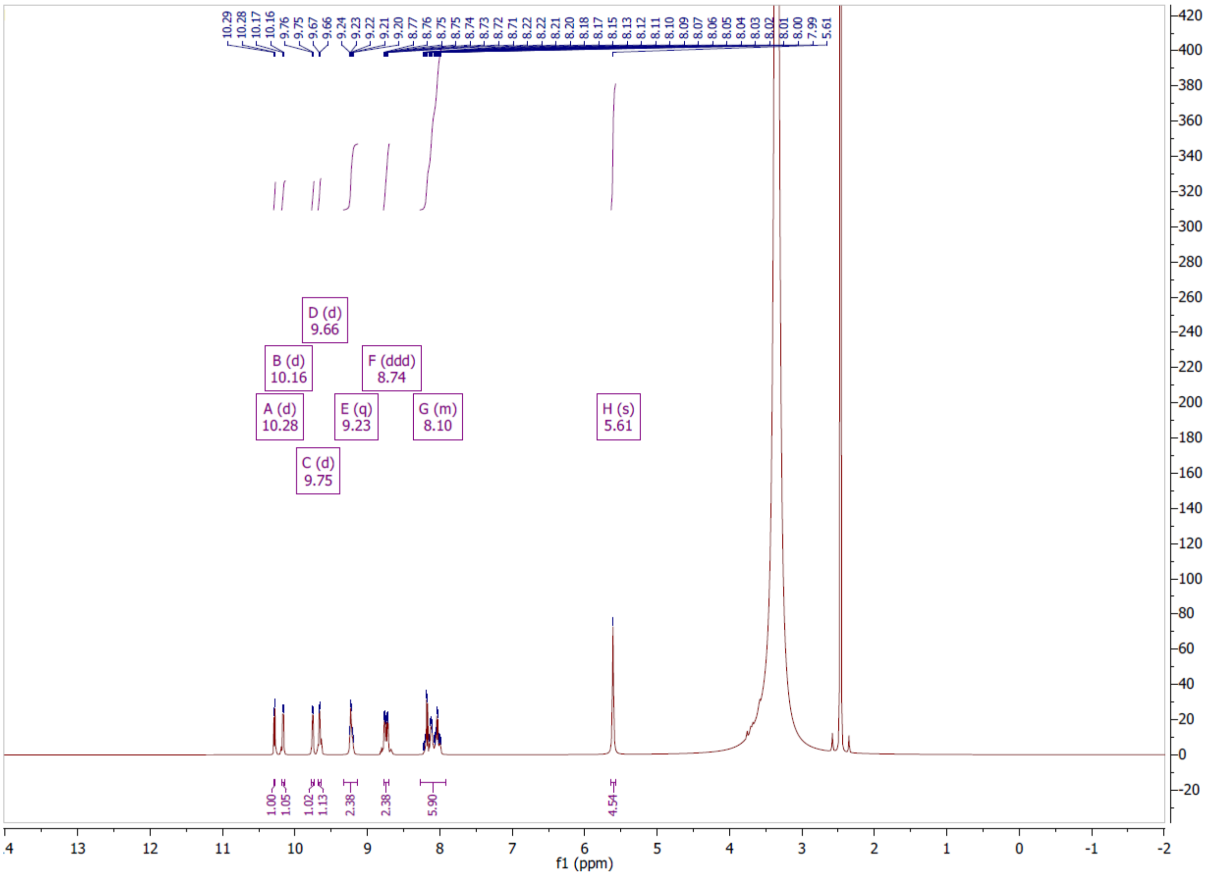

Figure S13. ^{1}H NMR spectrum of 2-q.

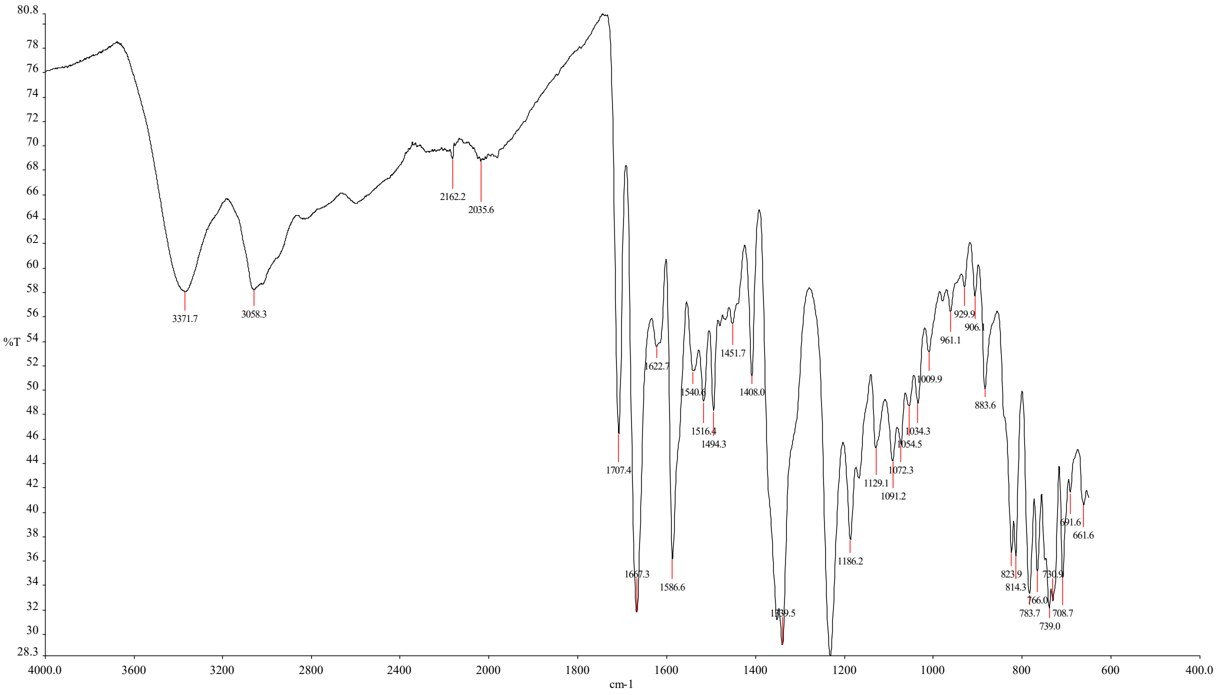

Figure S14. FT-IR spectrum of 2-q.

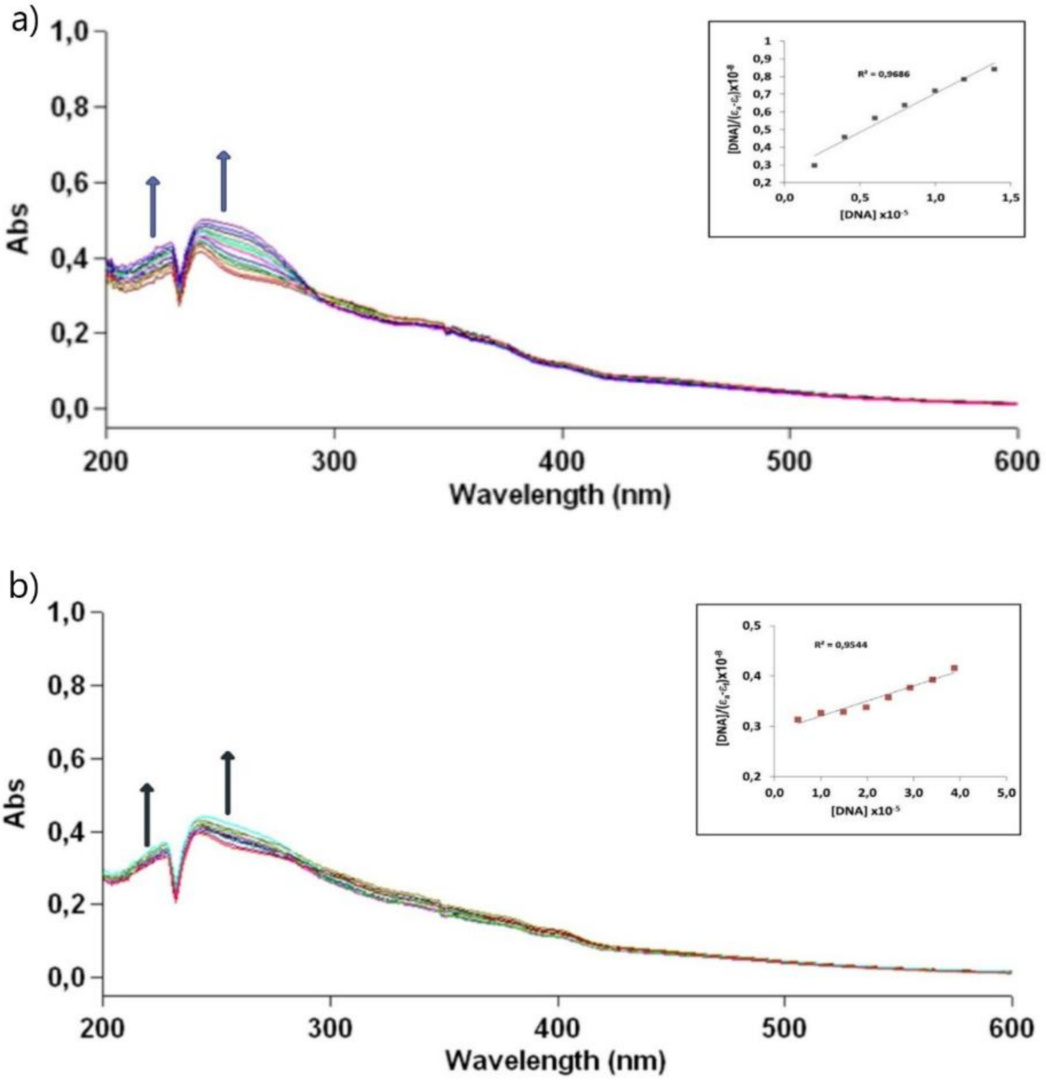

Figure S15. UV-Vis spectra of compound 1 with increasing amounts of ds-DNA (a) and G-DNA (b).

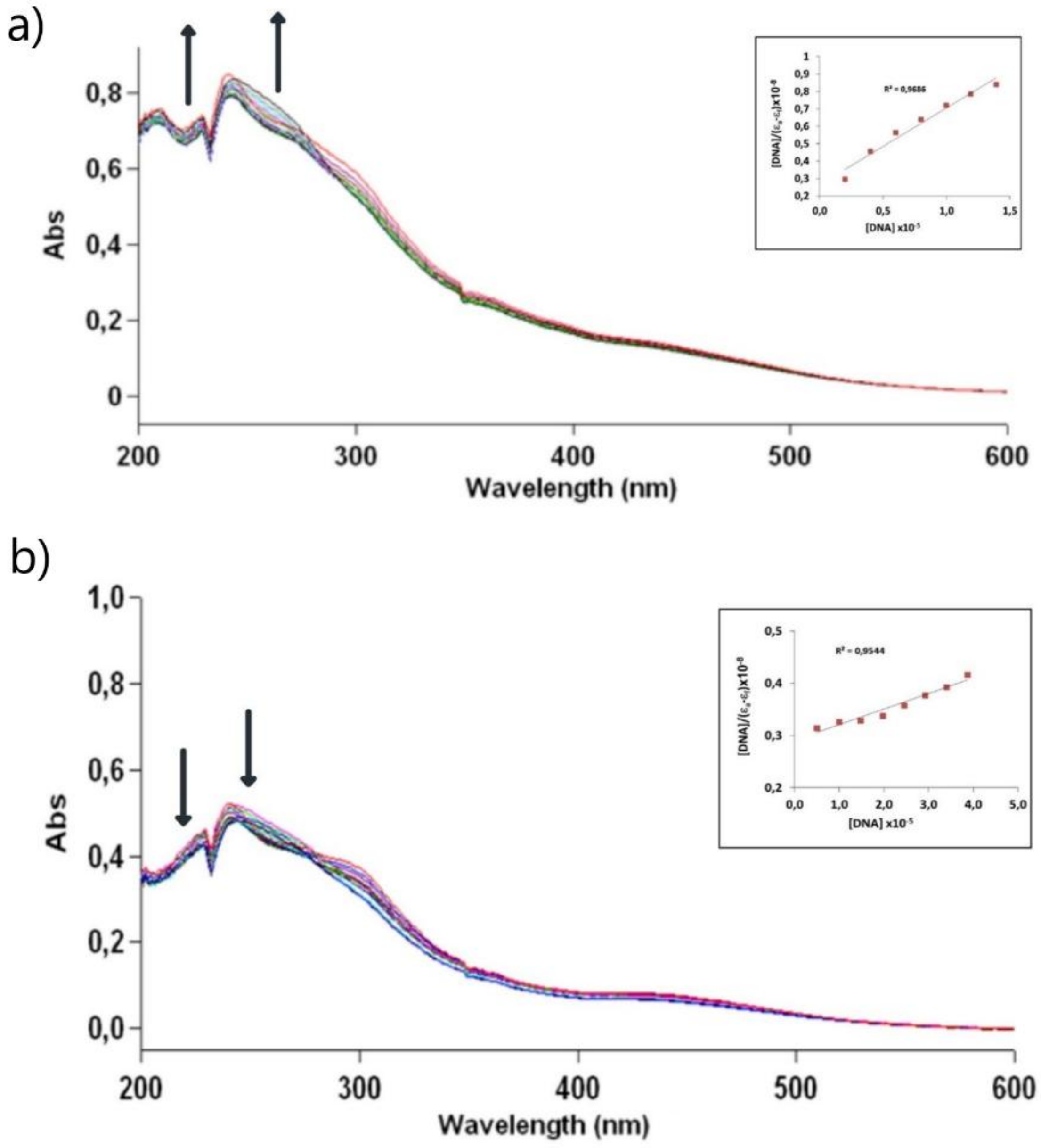

Figure S16. UV-Vis spectra of compound 2 with increasing amounts of ds-DNA (a) and G-DNA (b).

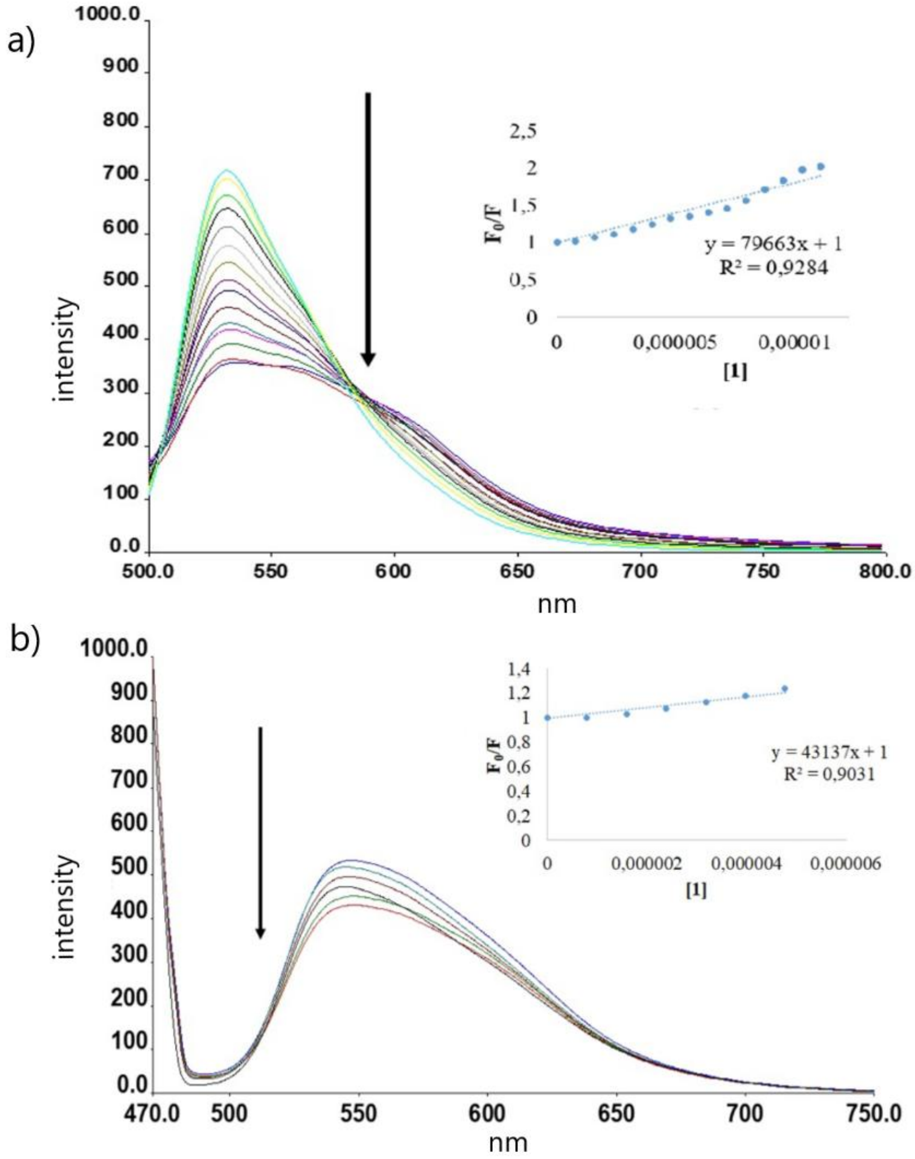

Figure S17. Emission spectrum obtained by adding increasing amounts of 1 onto the TO-dsDNA mixture (a) and the TO-G-quadruplex DNA mixture (b).

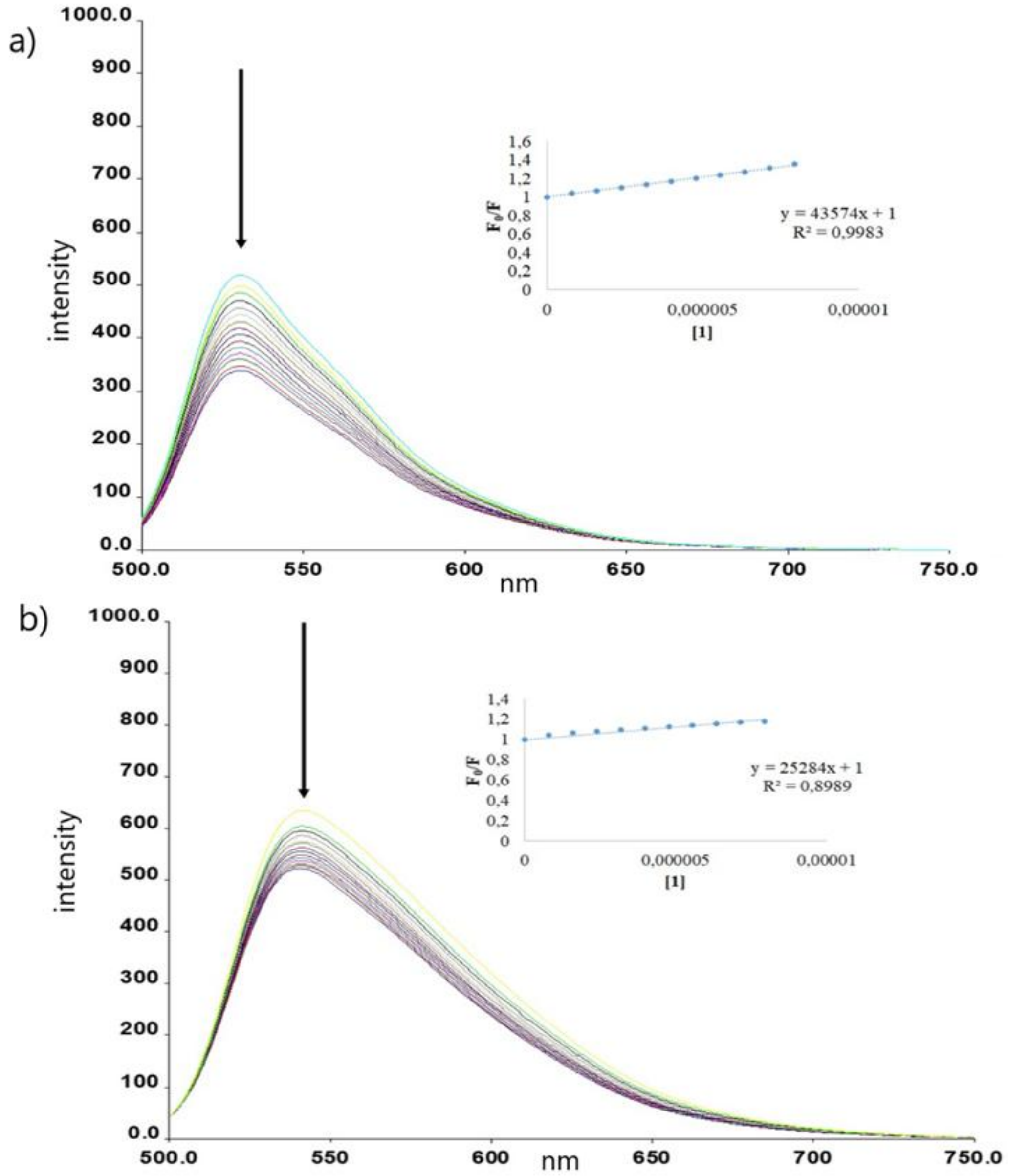

Figure S18. Emission spectrum obtained by adding increasing amounts of 2 onto the TO-dsDNA mixture (a) and the TO-G-quadruplex DNA mixture (b).

Table S1. DC_{50} values (μM) for ligand binding to double-stranded DNA (dsDNA) determined by FID assay (mean ± SD, n = 3).

Compounds	Repeats (μM)	Average ± SD	%RSD
1	12.1, 12.7, 12.6	**12.5 ± 0.32**	2.6%
1q	0.96, 1.02, 1.01	**1.00 ± 0.03**	3.0%
2	22.4, 23.3, 23.0	**22.9 ± 0.46**	2.0%
2q	1.60, 1.70, 1.68	**1.66 ± 0.05**	3.0%

Table S2. DC_{50} values (μM) for ligand binding to G-quadruplex DNA determined by FID assay (mean ± SD, n = 3).

Compounds	Repeats (μM)	Average ± SD	%RSD
1	22.8, 23.6, 23.2	**23.2 ± 0.40**	1.7%
1q	0.31, 0.34, 0.34	**0.33 ± 0.02**	4.8%
2	38.7, 40.1, 39.7	**39.5 ± 0.72**	1.8%
2q	2.95, 3.15, 3.14	**3.08 ± 0.11**	3.6%
